# Food Polysaccharides and Proteins: Processing, Characterization, and Health Benefits

**DOI:** 10.3390/foods13071113

**Published:** 2024-04-05

**Authors:** Liyuan Rong, Mingyue Shen, Yanjun Zhang, Hansong Yu, Jianhua Xie

**Affiliations:** 1 State Key Laboratory of Food Science and Resources, Nanchang University, Nanchang 330047, China; rongliyuan01@163.com (L.R.); shenmingyue1107@163.com (M.S.); 2 Spice and Beverage Research Institute, Chinese Academy of Tropical Agricultural Sciences, Wanning 571533, China; zhangyanjun0305@163.com; 3 College of Food Science and Engineering, Jilin Agricultural University, Changchun 130118, China; yuhansong@163.com

Natural macromolecular substances are prevalent in the organs of plants and animals, such as polysaccharides, resins, proteins, etc. With the progress of modern isolation technology as well as the development of enzyme engineering and modification technologies, the structural characteristics and functional activity of these natural components are gradually recognized, and they are widely applied in the food industry as important raw materials. Nowadays, the unsatisfactory characteristics of natural components, including their insolubility at lower temperatures, instability under certain conditions, viscosity change, and lack of functional properties, still limit their specialized mass production and downstream processing with high added value. Researchers in specific subjects from around the world devote themselves to further investigating the critical properties and mechanisms during the process, and a Special Issue titled “Food Polysaccharides, Starch, and Protein: Processing, Characterization, and Health Benefits” was launched for peers in related fields to exchange recent research progress on the modification of natural active substances and their functional activities.

Among these accepted manuscripts, various critical properties and mechanisms of natural macromolecular substances had been put forward, many focusing on the separation and purification of new protein and polysaccharide resources, as well as their changes in structure and nutritional function during processing. Polysaccharides are biomacromolecule carbohydrates wildly found in nature, consisting of multiple monosaccharide units connected by glycosidic bonds [1]. The advancement in isolation and identification techniques has led to a significant surge of interest and research in polysaccharides. The polysaccharides discovered in recent years have exhibited diverse biological activities, including anti-tumor, antioxidant, antibacterial, anti-inflammatory, and immunomodulatory effects [2,3]. Lu et al. [4] revealed that the Iljinskaja polysaccharide and Chinese yam polysaccharide (CYP) improved colitis symptoms in dextran sulfate sodium-induced mice by enhancing the production of IL-10, inhibiting cytokines (IL-1β and TNF), and reducing myeloperoxidase (MBO) activity. They also reduced the contents of lipopolysaccharide-binding protein (LBP) and endotoxin (ET) in serum and oxidative stress in the liver, promoting the expression of mucin MUC-2, ZO-1, and occluding to maintain the integrity of the intestine. The pretreatment of CYP relieved excessive oxidative stress by modulating the MAPK signaling pathway, with a corresponding preventive role against injury to the intestinal barrier [5].

Polysaccharides are applied to the development of special foods and packing materials due to their special composition and structure, such as dysphagia diet [6], 3D printing foods [7], and biodegradable food packaging [8]. The biological activities of natural polysaccharides extracted from various sources may not always meet satisfactory requirements and can even demonstrate suboptimal performance. The exploration and molecular modification of polysaccharide resources play a crucial role in promoting the utilization of polysaccharides in both the food and non-food industries [9,10]. Among the manuscripts accepted by the Special Issue, the exopolysaccharide (EPS) was successfully isolated and purified from probiotic *Enterococcus hirae* WEHI01. EPS was composed of I01-1, I01-2, I01-3, and I01-4, while I01-2 and I01-4 raised the viability and phagocytic function of macrophage cells, boosted NO generation, and encouraged the release of cytokines including TNF-α and IL-6 in RAW 264.7 macrophages. The main components of *Anoectochilus formosanus* polysaccharide (AFP) were galacturonic acid, glucose, and galactose, and AFP alleviated cyclophosphamide-induced immunosuppression and significantly improved the immunity of mice via stimulating the production of cytokines (IgA, IgG, SIgA, IL-2, IL-6, and IFN-γ). Obesity has become a global public health matter; it always causes a series of severe chronic diseases, such as metabolic syndrome, cardiovascular disease, type 2 diabetes, and neurodegenerative diseases [11]. In this Special Issue, the potential regulatory mechanisms of high-purity insoluble dietary fiber from soybean residue (HPSIDF) on hepatic fatty acid oxidation were investigated. The medium- and long-chain fatty acid oxidation in hepatic mitochondria was accelerated because HPSIDF effectively increased the levels of acyl-coenzyme A oxidase 1, malonyl coenzyme A, acetyl coenzyme A synthase, acetyl coenzyme A carboxylase, and carnitine palmitoyl transferase-1. HPSIDF supplementation significantly ameliorated co-occurring symptoms in high-fat diet-induced mice, including body weight gain, fat accumulation, dyslipidemia, and hepatic steatosis. As an important part of the innate immune system, the complement pathway is critical for identifying and clearing pathogens that rapidly react to defend the body against external pathogens. Xing et al. [12] reviewed the function and immunomodulatory mechanisms of complement component 1q (C1q), and they summarized the foods, including polysaccharides, with beneficial effects in neurodegenerative diseases via C1q and the complement pathway. For instance, *Artemisia annua* polysaccharides exhibited noteworthy efficacy in anticomplement activities through the classical pathway and alternative pathway, and *Prunella vulgaris* polysaccharides exhibited a potential value in addressing ailments correlated with the excessive activation of the complement system, while they could interact with C1q, exerting an influence on the C2, C3, C5, and C9 constituents of the complement system. 

The physicochemical properties of natural polysaccharides always need to be modified to meet the development of food science and technology, and there are considerable differences between the natural and modified polysaccharides [13]. This Special Issue highlighted that CYP and sulfated Chinese yam polysaccharides (SCYP) both promoted the proliferation of polysaccharide-degrading bacteria and facilitated the intestinal de-utilization of polysaccharides by producing more biomarkers of the gut microbiome. Differently, CYP regulated the gut microbiota by decreasing *Desulfovibrio* and *Sutterella* and increasing *Prevotella*, while SCYP changed the gut microbiota by decreasing *Desulfovibrio* and increasing *Coprococcus*, which reversed the microbiota dysbiosis caused by lipopolysaccharide. SCYP was more effective than CYP in reducing hepatic TNF-α, IL-6, and IL-1β secretion. Special dietary foods are specifically formulated to meet the nutritional needs of individuals with unique requirements. The dysphagia diet is a special eating plan for dysphagia patients, such as newborns, the elderly, and patients with postoperative muscle loss, neurological impairment, and Alzheimer’s disease [14]. In this Special Issue, high-methoxyl apple pectin was employed as the main component to improve the rheological behaviors for developing dysphagia-friendly fluidized alimentary matrices. The researchers prepared dysphagia foods using rice starch, perilla seed oil, and whey isolate protein and evaluated the positive effects of food supplements (vitamins, minerals, salt, and sugar) on the swallowing characteristics and rheological and textural properties of the prepared products. The work revealed that polysaccharides hold great potential for the special diet’s application, and the combination of the nutritional functional activity and the sensory physical properties could be the research priorities in the following research work [15,16]. As a macromolecular polysaccharide aggregate, insoluble dietary fiber will also be focused on in the Special Issue, and the developing status of technologies for the separation and extraction of single components in insoluble dietary fiber will be reviewed, aiming to expand their application in the food and non-food fields.

Proteins play a major role in human life, serving as the basic structural material of the body as well as biochemical catalysts and regulators of genes [17,18]. The soybean trypsin inhibitors caused structural damage and secretory dysfunction of the pancreas, increasing lipid peroxidation and injuring the enzymatic and non-enzymatic antioxidant defenses in the soybean trypsin inhibitor diet-fed mice. Proteins could be denatured, altering their physiochemical properties and functions during thermal or non-thermal treatment. The denaturation of proteins is a prevalent occurrence in processing, and comprehending and harnessing the principles and mechanisms behind protein denaturation holds immense significance [19,20]. Among the papers published in the Special Issue, suitable cavitation jet treatment (CJT) improved the food proteins’ functionalities by adjusting the structural and functional features of solvable oxidized soybean protein accumulations. The CJT at a short treatment time destroyed the core aggregation skeleton of soybean protein insoluble aggregates and transferred the insoluble aggregates into soluble aggregates. The prolonged CJT reaggregated the soluble oxidized aggregates through an anti-parallel intermolecular β-sheet, resulting in a lower emulsification activity index (EAI) and emulsification stability index (ESI) and a higher interfacial tension. The effects of hydrothermal treatment on the structure and functional properties of quinoa protein isolate (QPI) were studied. The secondary and tertiary structures of QPI were significantly changed after hydrothermal treatment, which accounted for the hydrothermal treatment. QPI exhibited a better functional property, while the suitable hydrothermal treatment improved its water-holding and oil-holding capacity, emulsifying activity, emulsion stability, and solubility [21]. As the main macromolecules concerned in this Special Issue, proteins and polysaccharides were also studied in innovative combinations. Water-unextractable arabinoxylan (WUAX) improved the textural property of flour by interacting with starch or gluten and investigating its structure-activity relationship [22]. WUAX increased the free sulfhydryl of gliadins and glutenins, inhibiting the formation of covalent bonds. WUAX decreased the β-sheet content and increased the β-turn prevalence of gliadins and glutenins. Differently, the WUAX decreased the contents of α-helixes and β-sheets for glutenins, and it did not significantly change these values of gliadins [22]. Consequently, WUAX could cause a quality deterioration of gluten by weakening the structure of the gliadins and glutenins.

In summary, the manuscripts published in this Special Issue have explored various innovative bioactive macromolecular resources and studied their structural characteristics, bioactivity, and nutritional function properties, as well as their application potential in the food industry. These results are helpful for colleagues to understand the health benefits of homologous resources in medicine and food and the characterization methods in the discovery process. The information presented in this Special Issue will promote the widespread use of macromolecules such as polysaccharides and proteins in the food industry. 
